# Reduced Upper Limb Recovery in Subcortical Stroke Patients With Small Prior Radiographic Stroke

**DOI:** 10.3389/fneur.2019.00454

**Published:** 2019-05-08

**Authors:** Matthew A. Edwardson, Li Ding, Caron Park, Christianne J. Lane, Monica A. Nelsen, Steven L. Wolf, Carolee J. Winstein, Alexander W. Dromerick

**Affiliations:** ^1^Department of Neurology, Georgetown University, Washington, DC, United States; ^2^Department of Rehabilitation Medicine, Center for Brain Plasticity and Recovery, Georgetown University and MedStar National Rehabilitation Hospital, Washington, DC, United States; ^3^Division of Biostatistics, Department of Preventive Medicine, Keck School of Medicine, University of Southern California, Los Angeles, CA, United States; ^4^Division of Biokinesiology and Physical Therapy, Herman Ostrow School of Dentistry, University of Southern California, Los Angeles, CA, United States; ^5^Physical Therapy Division, Departments of Rehabilitation Medicine, Medicine and Cell Biology, Emory Rehabilitation Hospital, Emory University School of Medicine, Atlanta, GA, United States; ^6^VA Center on Visual and Neurocognitive Rehabilitation, Decatur, GA, United States; ^7^Department of Neurology, Keck School of Medicine, University of Southern California, Los Angeles, CA, United States

**Keywords:** subcortical infarction, stroke rehabilitation, occupational therapy, upper extremity paresis, magnetic resonance imaging, internal capsule, basal ganglia

## Abstract

**Background:** Research imaging costs limit lesion-based analyses in already expensive large stroke rehabilitation trials. Despite the belief that lesion characteristics influence recovery and treatment response, prior studies have not sufficiently addressed whether lesion features are an important consideration in motor rehabilitation trial design.

**Objective:** Using clinically-obtained neuroimaging, evaluate how lesion characteristics relate to upper extremity (UE) recovery and response to therapy in a large UE rehabilitation trial.

**Methods:** We reviewed lesions from 297 participants with mild-moderate motor impairment in the Interdisciplinary Comprehensive Arm Rehabilitation Evaluation (ICARE) study and their association with motor recovery, measured by the UE Fugl-Meyer (UE-FM). Significant lesion features identified on correlational and bivariate analysis were further analyzed for associations with recovery and therapy response using longitudinal mixed models.

**Results:** Prior radiographic stroke was associated with less recovery on UE-FM in participants with motor impairment from subsequent subcortical stroke (−5.8 points) and in the overall sample (−3.6 points), but not in participants with cortical or mixed lesions. Lesion volume was also associated with less recovery, particularly after subcortical stroke. Every decade increase in age was associated with 1 less point of recovery on UE-FM. Response to specific treatment regimens varied based on lesion characteristics. Subcortical stroke patients experienced slightly less recovery with higher doses of upper extremity task-oriented training. Participants with cortical or mixed lesions experienced more recovery with higher doses of usual and customary therapy. Other imaging features (leukoaraiosis, ischemic vs. hemorrhagic stroke) were not significant.

**Conclusions:** ICARE clinical imaging revealed information useful for UE motor trial design: stratification of persons with and without prior radiographic stroke may be required in participants with subcortical stroke, the majority of motor rehabilitation trial participants. Most of the prior radiographic strokes were small and cortically-based, suggesting even minor prior brain injury remote to the acute stroke lesion may limit spontaneous and therapy-related recovery. Lesion location may be associated with response to different therapy regimens, but the effects are variable and of unclear significance.

## Introduction

Identifying factors that contribute to variability in motor recovery and response to therapy are important considerations to enhance motor rehabilitation trial design. Stroke patients generally experience some degree of recovery from their motor deficits over the first several months following injury (1, 2). At least in small studies of highly refined samples, some of the variability in motor recovery can be explained by particular stroke lesion characteristics (3). For example, evidence from studies using dedicated, research quality MRI suggests that damage to the corticospinal tract is associated with poor motor recovery (4, 5). Lesion volume may be related to worse recovery for particular stroke locations or depths, including the subcortical area (3, 6). The neuroimaging features contributing to response to rehabilitation therapy remain understudied. Small investigations in chronic stroke patients suggest less damage to the corticospinal tract on research grade MRI (7–9) may predict a more favorable treatment response.

Large multicenter trials offer the opportunity to examine whether lesion features affect recovery and response to therapy, but most prior stroke rehabilitation trials did not characterize stroke lesions on neuroimaging (10–12). This is largely because of the cost of dedicated, research-grade MRI for already expensive rehabilitation trials and the assessment burden in physically disabled individuals. Methods to extract lesion features from MRI or CT obtained during routine clinical care do exist (13), and may provide important information with regard to recovery and response to therapy. A large rehabilitation trial in patients with lower extremity impairment used clinical neuroimaging and found that stroke lesions in the basal ganglia or prior stroke on neuroimaging resulted in less improvement in walking speed at 1 year (14). No one has assessed the neuroimaging features related to motor recovery and response to therapy from a large multicenter stroke rehabilitation trial focused on the upper extremity. In addition, no prior studies assessing lesion features in the acute-subacute timeframe (<6 mo. post-stroke) included a control group receiving lesser amounts of therapy. The tremendous spontaneous recovery that occurs during this acute-subacute stage and lack of appropriate controls have made it difficult to determine whether lesion features truly impact therapy-related gains.

The design of the Interdisciplinary Comprehensive Arm Rehabilitation Evaluation (ICARE) trial provided an opportunity to address some of these issues. ICARE was large-scale, included two dose matched treatments that differed in motor training paradigms, and had a usual and customary care group that received less motor training. ICARE procedures did not involve neuroimaging, but we systematically collected all clinical neuroimages for later study. We assessed whether lesion features identified on these routine images could be useful in rehabilitation trial design. In this exploratory study, lesion characteristics were studied to see if they could explain variance in motor recovery and response to therapy in a large group of patients using longitudinal mixed regression models.

## Materials and Methods

### Participants

ICARE was a multicenter, randomized controlled trial testing the effects of two forms of upper extremity therapy in the subacute phase (median 42, range 14–138 days) following ischemic or hemorrhagic stroke: a principle-based, task-oriented therapy approach (Accelerated Skill Acquisition Program, ASAP), and a dose equivalent usual and customary care therapy program (DEUCC). It also examined the question of dose by including a third monitoring-only usual and customary care therapy program (UCC). Following institutional review board approval at each site, participants were recruited from 7 separate rehabilitation hospitals in Atlanta, Washington DC, and the greater Los Angeles area. All participants gave written informed consent in accordance with the Declaration of Helsinki. The main inclusion/exclusion criteria were as follows: *inclusion criteria*—ischemic or hemorrhagic stroke, arm or hand hemiparesis [Upper Extremity Fugl Meyer (UE-FM) 19–58], some active finger extension, age ≥21; *exclusion criteria*—prior neurologic condition that may affect motor response, absent upper extremity sensation, neglect (score > 3 on Mesulam Unstructured), and >6 outpatient occupational therapy sessions prior to enrollment. ICARE hypothesized that the ASAP group would have superior motor recovery when compared to each of the DEUCC and UCC groups. There was no difference in the study's primary outcome measure, the change in the log-transformed Wolf Motor Function Test time score 1 year post-randomization, across the three groups (15).

### Neuroimaging Analysis

All ICARE participants with clinical MRI or CT available as well as a stroke lesion identified on neuroimaging were included in the study. Neuroimages for each participant were uploaded into a custom web-based imaging viewer (16). Ischemic stroke lesions were identified using diffusion-weighted imaging (DWI) if ≤7 days from symptom onset, fluid-attenuated inversion recovery (FLAIR) if >7 days, or CT if MRI was unavailable. Intracranial hemorrhage (ICH) lesions were identified using CT; gradient echo sequences (GRE) were used when CT was unavailable. Lesion characteristics including lesion type (ischemic or hemorrhagic), planimetric volume, location, depth (cortical/subcortical/both), presence of old strokes, and degree of leukoaraiosis were determined.

Lesion location was determined by overlaying vascular territory templates (13). In this approach, templates with outlined subdivisions representing all of the vascular territories found at each particular axial section of the brain are overlaid on top of each corresponding axial slice. The predominant area is the vascular territory subdivision located in the 3-dimensional center of the lesion. The predominant area location is not synonymous with the anatomic vascular territory for the entire stroke lesion. For example, a stroke centered in the anterior choroidal or insular template region may represent a larger middle cerebral artery stroke [see (13) for template example and further details]. Participants with the predominant area categorized as either anterior choroidal (ACH) or basilar were chosen for location analysis because these regions overlap the corticospinal tract (CST). We did not perform more specific analysis of injury to the CST, such as tractography or lesion load analysis, due to the mix of MRI and CT and having only clinical as opposed to research quality neuroimaging available. This lack of tractography data has important implications, as some of the lesions centered in the ACH and basilar regions may not affect the CST, or affect it only minimally.

Lesion depth was determined qualitatively by visualizing the lesion in the imaging viewer and was defined as: cortical—superficial to the basal ganglia; subcortical—structures deep to the cortical area including the basal ganglia, thalamus, and brainstem; or mixed—stroke lesion spanning both the cortical and subcortical regions. Differences in axial slice thickness for individual imaging scanners were accounted for in planimetric lesion volume calculations. Lacunar strokes were defined as subcortical lesions ≤2 cm in axial diameter (17). Old strokes were defined as lesions >1 cm in diameter on FLAIR or CT, non-contiguous with the ventricular border. Leukoaraiosis was graded according to the Age-Related White Matter Disease Scale (ARWMC) (18). Mild, moderate, and severe leukoaraiosis corresponded to a maximum score of 1, 2, or 3, respectively in any of the 5 ARWMC regions. Two vascular neurologists (ME and AD), blinded to treatment group assignment and motor scores, independently read 25 scans using DWI; ME read all remaining scans. There was good interrater reliability (IRR) between the two reviewers for predominant area (*Kappa Statistic* = 0.98) and planimetric volume (92% of reads within 95% confidence interval on Bland-Altman plot, with limits of agreement ranging from −51.4 to 31.8% and more discrepancy between readers at smaller lesion volumes, see Supplementary Figure 1). Although we did not test across imaging modality, others have demonstrated good IRR for lesion characterization with both MRI and CT (19, 20).

### Outcome Measures

Outcome measures were obtained at baseline as well as immediately post-treatment at 4, 6 months, and 1 year post-randomization. The UE-FM was chosen as the primary outcome measure as opposed to a functional scale like the Wolf Motor Function Test used in the primary outcome paper in order to capture impairment rather than disability (21) and facilitate comparisons with the prior recovery and response to therapy literature. While one could argue that functionality is more important to the patient, we wished to capture return of pre-stroke movement patterns as opposed to compensation.

### Statistical Analysis

To assess the strength of the relationship between various stroke lesion neuroimaging characteristics and recovery [defined as the change (Δ) in UE-FM from baseline—post-intervention, baseline−6 months, and baseline−12 months], we first performed unadjusted Spearman correlations for continuous variables and Chi-squared tests for categorical variables. For the bivariate analysis, the Wilcoxon rank-sum test was used to compare continuous variable median between two groups, and the Kruskal–Wallis test with Bonferroni correction for more than two groups. Correlational analysis was performed between lesion volume and recovery at different lesion depths (subcortical, cortical, and mixed) and locations (anterior choroidal and basilar). In the bivariate analysis, recovery was assessed for the categorical variables—treatment group assignment, lesion depth, predominant area (lesion location, including anterior choroidal, basilar, and all other), old stroke either clinically or radiographically, stroke type (hemorrhagic or ischemic), and leukoaraiosis. The significance level was set to 0.05. Variables related to recovery in the correlational or bivariate analysis were then carried forward for assessment with linear mixed effects modeling.

To examine the effect of lesion volume on ΔUE-FM across the 12 months of the study allowing for each time segment (baseline—post-intervention, baseline-−6 months, and baseline-−12 months) to have a different slope accounting for non-linear changes across time we performed longitudinal linear mixed effects modeling. All post-treatment time points were compared to baseline. UE-FM was centered at the overall mean so as to make the intercept in the models meaningful to interpretation. All models were adjusted *a priori* for treatment group assignment, presence of old stroke on neuroimaging, and age in years (centered at 60); adjustment for baseline UE-FM is inherent in the models.

Models were built with increasing complexity, first looking at main effect of lesion volume within the model, adjusting for the 3 *a priori* covariates described above, then adding in interactions of volume by group assignment to determine whether the lesion volume moderated the treatment effect on UE-FM. Following this approach, we examined the longitudinal trends and interactions of time with volume to determine whether lesion volume had a consistent effect across the year of follow-up, or if the effects were different each time post-stroke. We demonstrated in our previous work (22) that there are different trends in recovery by groups, therefore a group x time model interaction was also included. Comparison of model fits were made by examining fit indices (Akaike's information criterion, Bayesian information criterion), and comparing differences in−2Ln Likelihood of nested models to see if adding complexity also added in understanding. The difference in−2Ln Likelihood of two nested models follows a χ^2^ distribution with df being the difference in the df of the two models (α = 0.05 for these comparisons). The best fitting model for each subgroup was reported and consists of the most parsimonious model determined by overall model fit as well as individual predictors showing statistical importance (*p* < 0.10) beyond the *a priori* covariates. This choice of α = 0.10 for specific variables within the model was used as we acknowledge that these analyses are exploratory and therefore there may be insufficiently powered to detect significant effects within these models. These were only assessed after the best overall model fit was determined at α = 0.05. All analyses were performed using SPSS version 24 (IBM Corp., Armonk, NY).

## Results

Two-hundred and ninety-seven out of three-hundred and sixty-one ICARE participants had interpretable neuroimaging available. Among those with uninterpretable neuroimaging, eight participants had a CT on the same date as the stroke with no stroke identified. Twenty other participants had no stroke identifiable on neuroimaging, in most cases because the scan date was much later than the stroke date making it difficult to identify where or which lesion was acute. Eighteen participants had insufficient imaging—wrong organ system or the brain imaging was missing axial slices. The remaining 18 participants with missing data had no imaging available. There was no significant difference between included and not included participants on UE-FM at baseline (mean ± SD, 41.6 ± 9.5 vs. 41.8 ± 8.7, *p* = 0.87), post-intervention (50.1 ± 10.5 vs. 49.2 ± 9.9, *p* = 0.58), 6 mo. (51.1 ± 10.2 vs. 48.7 ± 10.5, *p* = 0.13), and 1 year (53.3 ± 10.5 vs. 50.4 ± 12.8, *p* = 0.09).

**Figure 1:**
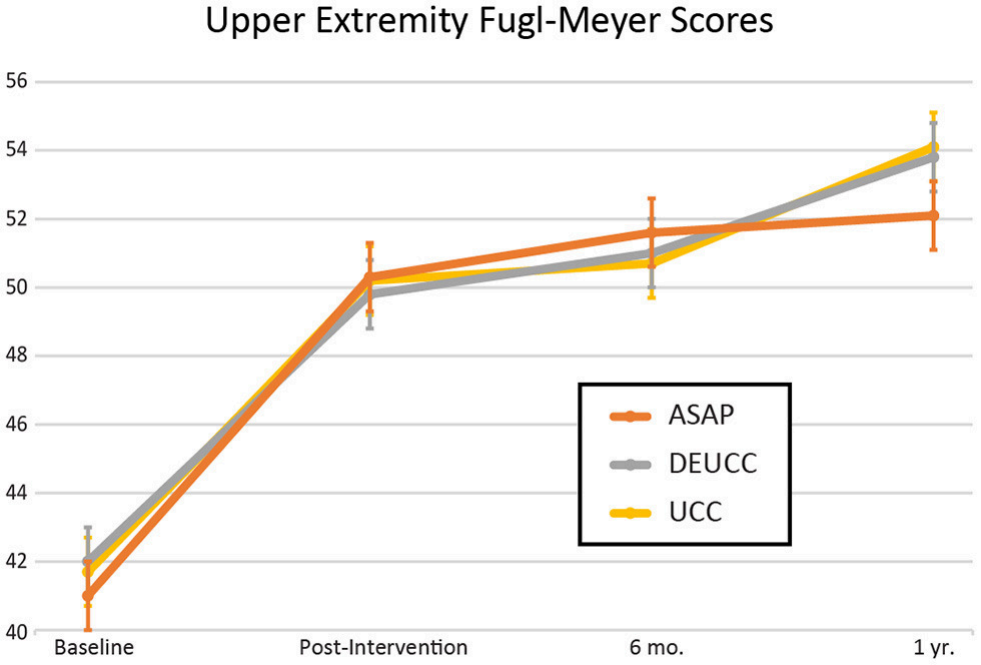
Upper extremity Fugl-Meyer scores over time for the 3 treatment groups in the ICARE clinical trial. Error bars represent standard deviation. ASAP, Accelerated Skill Acquisition Program; DEUCC, Dose Equivalent Usual and Customary Care; UCC, Usual and Customary Care.

Patient characteristics for the 297 included in the study are reported in Table 1. The mean total number of treatment hours for participants randomized to ASAP, DEUCC, and UCC was 27.9, 22.0, and 11.2, respectively. There was great variation in lesion volume with significant right skew (range 0.1–213 mL, median 2.4 mL), hence the natural log of the volume was used in all statistical tests. There were no group differences in stroke volume across the 3 treatment groups (*p* = 0.82). UE-FM improved asymptotically over time during the study period as follows: baseline (M+SD = 42 ± 10), post-intervention (50 ± 11), 6 months (51 ± 10), and 12-months (53 ± 11). There were no differences in UE-FM across groups at any time point (Figure 1, all *p*'s > 0.40), in agreement with the previously reported ICARE primary outcome data (15). Table 2 reports details of the stroke lesions in this sample. For the 258 ischemic strokes, DWI was used in lesion analysis for 186, FLAIR for 19, and CT for 53; for the 39 primary ICH participants, CT was used for 31 and GRE for 8. Forty-six percent of the overall sample had lacunar strokes and 39% of the ischemic stroke patients had large vessel infarcts, as reported previously (13). Among the patients with subcortical stroke, 62% were lacunar. Forty-four participants had prior strokes on neuroimaging as compared to only 15 reported by clinical history. Of the participants with prior strokes on neuroimaging, only 3 would be considered lacunar infarctions (subcortical, <2 cm diameter). Most of the prior radiographic strokes were small (median 2.1 mL) and cortical (86%).

**Table 1:** Clinical characteristics for ICARE neuroimaging participants.

Clinical characteristics^*^	**Total (*N* = 297)**
Age, mean (SD), year	60.7 (12.2)
Sex, No. (%)	
Male	165 (56%)
Female	132 (45%)
Time from stroke onset to randomization, median (range), days	42 (14-138)
Time from stroke onset to neuroimaging, median (IQR, range), days	1 (0–3, 0–127)
Stroke Type, No. (%)	
Ischemic	258 (87%)
Hemorrhagic	39 (13%)
Stroke Side, No. (%)	
Left	122 (41%)
Right	172 (58%)
Midline	3 (1%)
Old Stroke by Clinical History, No. (%)	
Yes	15 (5%)
No	282 (95%)

* Percentages may not total 100 due to rounding.

**Table 2:** Stroke lesion neuroimaging characteristics.

**Lesion characteristics**	**Total (*N* = 297)**	**ASAP (*N* = 95)**	**DEUCC (*N* = 103)**	**UCC (*N* = 99)**
Planimetric volume, median (IQR), mL	2.4 (1–12.8)	2 (0.9–14.3)	2.5 (1.2–12.6)	2.8 (1–13.4)
**DEPTH, NO. (%)**				
Cortical	44 (15%)	15 (16%)	16 (16%)	13 (13%)
Subcortical	172 (58%)	53 (56%)	61 (59%)	58 (59%)
Mixed (cortical plus subcortical)	81 (27%)	27 (28%)	26 (25%)	28 (28%)
LOCATION (PREDOMINANT AREA), NO. (%)^*^				
Anterior cerebral artery (ACA)	19 (6%)	7 (7%)	8 (8%)	4 (4%)
Middle cerebral artery (MCA)	58 (20%)	17 (18%)	17 (17%)	24 (24%)
Heubner's artery (HA)	0	0	0	0
Lenticulostriates (LS)	13 (4%)	6 (6%)	4 (4%)	3 (3%)
Insular branches (IB)	10 (3%)	3 (3%)	4 (4%)	3 (3%)
Anterior choroidal (ACH)	109 (37%)	35 (37%)	35 (34%)	39 (39%)
Thalamoperforating (THP)	14 (5%)	4 (4%)	5 (5%)	5 (5%)
Posterior cerebral artery (PCA)	2 (1%)	0	2 (2%)	0
Basilar artery (BA)	67 (23%)	21 (22%)	28 (27%)	18 (18%)
Vertebral artery (VA)	5 (2%)	2 (2%)	0	3 (3%)
**OLD STROKE BY NEUROIMAGING, NO. (%)**				
Yes	44 (15%)	13 (14%)	16 (16%)	15 (15%)
No	253 (85%)	82 (86%)	87 (84%)	84 (85%)
Old stroke by neuroimaging volume, median (IQR), mL^†^	2.1 (0.5–9)	5.4 (2.4–15.5)	1.1 (0.5–4)	1 (0.5–6.9)
**LEUKOARAIOSIS^‡^, NO. (%)**				
None	104 (35%)	36 (38%)	37 (36%)	31 (31%)
Mild	129 (43%)	36 (38%)	49 (48%)	44 (44%)
Moderate–severe	64 (22%)	23 (24%)	17 (17%)	24 (24%)

ASAP, Accelerated Skill Acquisition Program; DEUCC, Dose Equivalent Usual and Customary Care; UCC, Usual and Customary Care.

* Predominant area reflects the 3-dimensional center of the stroke lesion (13).

† Estimated using the ABC/2 method (23).

‡ Graded according to the Age-Related White Matter Disease Scale (ARWMC) (18). Mild, moderate, and severe correspond to a maximum score of 1, 2, or 3, respectively in any of the 5 ARWMC regions.

### Correlational and Bivariate Analysis

To investigate features on clinical neuroimaging related to recovery suitable for further analysis in mixed models, we performed correlational and bivariate analysis. Correlational analysis (Table 3) revealed a significant association between lesion volume and change in the UE-FM from baseline to 1 year in the subcortical group (*R* = 0.24, *P* = 0.004), but not in the overall sample (*R* = 0.04, *P* = 0.48). Bivariate analysis (Table 4) showed that participants with chronic stroke lesions on neuroimaging exhibited less recovery based on the change in UE-FM from baseline to post-intervention (5.8 ± 6.1 vs. 9.1 ± 7.8, *P* = 0.02) and baseline to 6 months (6.6 ± 6.9 vs. 10 ± 8.1, *P* = 0.02), but not baseline to 1 year (9.6 ± 9.8 vs. 11.8 ± 8.8, *P* = 0.18). In summary, the correlational and bivariate analyses revealed associations between stroke lesion volume and ΔUE-FM for particular lesion depths and time points, and between old stroke and ΔUE-FM. These variables were therefore chosen for further analysis using longitudinal linear mixed effects modeling.

**Table 3:** Association between lesion volume and upper extremity Fugl-Meyer score by lesion depth and location.

	**All participants** ** (*****n*** **=** **297)**		**Subcortical** ** (*****n*** **=** **172)**		**Cortical** ** (*****n*** **=** **44)**		**Mixed** ** (*****n*** **=** **81)**		**ACH** ** (*****n*** **=** **109)**		**Basilar** ** (*****n*** **=** **67)**	
	***R***	***P*-value**	***R***	***P*-value**	***R***	***P*-value**	**R**	***P*-value**	***R***	***P*-value**	***R***	***P*-value**
**Change in UE-FM**
Baseline to post-intervention	0.01	0.87	0.05	0.54	−0.04	0.79	0.06	0.61	0.01	0.9	6.30E-04	1
Baseline to 6 month	0.05	0.45	0.14	0.09	−0.04	0.80	0.06	0.62	0.1	0.35	0.12	0.37
Baseline to 1 year	0.04	0.48	0.24	0.004	−0.16	0.35	0.09	0.48	0.12	0.25	0.2	0.14

*ACH, anterior choroidal artery; Mixed, stroke lesion spanning the cortical and subcortical regions; R, Spearman's correlation coefficient; UE-FM, Upper Extremity Fugl-Meyer Score*.

**Table 4:** Association between change in upper extremity Fugl-Meyer scores and clinical/neuroimaging characteristics.

	**Change in UE-FM score**											
	**Baseline to post-intervention**				**Baseline to 6 months**				**Baseline to 1 year**			
	**N**	**Mean**	**Std**	***P*-value**	***N***	**Mean**	**Std**	***P*-value**	***N***	**Mean**	**Std**	***P*-value**
Treatment group				0.46				0.28				0.63
ASAP	84	9.44	8.29		83	10.62	8.31		83	10.72	9.15	
DEUCC	87	8.26	6.82		87	9.42	7.42		92	11.91	8.56	
UCC	80	8.06	7.95		74	8.61	8.34		78	11.85	9.25	
Lesion depth				0.52				0.17				0.34
Cortical	37	7.05	6.72		34	7.21	7.40		36	9.53	7.93	
Subcortical	146	8.73	8.23		142	9.88	8.57		147	11.99	9.68	
Both	68	9.13	6.95		68	10.16	6.93		70	11.50	7.76	
Predominant area^*^				0.19				0.33				0.20
ACH	93	9.73	8.68		89	10.43	8.81		94	12.64	10.06	
Basilar	55	8.10	7.30		56	9.79	7.48		57	11.70	8.23	
All other	103	7.83	6.85		99	8.71	7.54		102	10.34	8.17	
Radiographic old stroke				0.02				0.02				0.18
Yes	35	5.80	6.12		32	6.56	6.87		35	9.62	9.80	
No	216	9.05	7.84		212	10.04	8.09		218	11.80	8.80	
Clinical old stroke				0.27				0.16				0.40
Yes	10	6.10	7.45		10	6.20	6.92		11	9.55	7.98	
No	241	8.70	7.70		234	9.73	8.04		242	11.59	9.00	
Stroke type				0.49				0.68				0.49
Hemorrhagic	29	7.66	9.88		28	9.00	9.25		30	10.43	8.45	
Ischemic	222	8.10	7.38		216	9.66	7.86		223	11.65	9.03	
Leukoaraiosis^†^				0.13				0.38				0.13
None	88	8.17	7.06		86	9.48	7.82		90	11.02	8.31	
Mild	110	9.62	7.54		111	10.22	8.19		106	12.75	9.18	
Moderate/severe	53	7.17	8.81		47	8.29	7.96		57	9.94	9.33	

* Predominant area reflects the 3-dimensional center of the stroke lesion (13).

† Graded according to the Age-Related White Matter Disease Scale (ARWMC) (18). Mild, moderate, and severe correspond to a maximum score of 1, 2, or 3, respectively in any of the 5 ARWMC regions.

The correlational and bivariate analyses were also notable for many clinical neuroimaging features that do not relate to motor recovery. Stroke location in the anterior choroidal or basilar template regions were not associated with motor recovery, nor was the presence of leukoaraiosis or distinction between ischemic or hemorrhagic stroke.

### Longitudinal Mixed Effects Models

To investigate the relationship between the significant clinical neuroimaging variables and recovery and response to therapy after adjusting for *a priori* covariates we constructed mixed effects models. Mixed effects results are reported for the overall sample, the subcortical lesion depth and a combination of the cortical + mixed lesion depth groups (Table 5). The cortical + mixed lesion depth results were included in order to contrast the results of the subcortical group with all remaining participants. The median planimetric volume for all participants, subcortical, and cortical + mixed was 2.4, 1.2, and 15.7 mL, respectively. There was a trend toward a difference between UE-FM scores in the subcortical and cortical + mixed group at baseline (mean ± SD, 40.7 ± 9.7 vs. 42.9 ± 9.1, *p* = 0.06), but not post-intervention (49.6 ± 10.7 vs. 50.8 ± 10.2, *p* = 0.37), 6 mo. (50.8 ± 10.5 vs. 51.5 ± 9.9, *p* = 0.59, or 1 year (53.1 ± 10.6 vs. 53.6 ± 10.3, *p* = 0.75). No mixed models are reported for the anterior choroidal and basilar groups since they did not reach statistical significance in the correlational/bivariate analysis.

**Table 5:** Longitudinal mixed regression analyses.

**Variable**	**Change in upper extremity Fugl-Meyer**	**Standard error**	***P* Value**
**ALL PARTICIPANTS (*****N*** **=** **297)**			
Intercept	−4.53	0.84	<0.01
Age (years)	−0.09	0.03	<0.01
Log planimetric volume (mL)	−0.84	0.32	0.01
Treatment group (vs. UCC)			0.31
ASAP	−1.12	0.94	*A vs. U = 0.24* *A vs. D = 0.78*
DEUCC	−1.37	0.95	*D vs. U = 0.15*
Old stroke on neuroimaging	−3.64	0.90	<0.01
Post-Intervention	8.58	0.85	<0.01
6 months	9.53	0.85	<0.01
12 months	11.75	0.85	<0.01
Group × volume			0.09
**SUBCORTICAL (*****N*** **=** **172)**			
Intercept	−3.97	0.95	<0.01
Age (years)	−0.10	0.03	<0.01
Log planimetric volume (mL)	−3.90	0.64	<0.01
Treatment group (vs. UCC)			0.01
ASAP	−2.99	1.01	*A vs. U = <0.01* *A vs. D = 0.69*
DEUCC	−2.60	0.99	*D vs. U = 0.01*
Old stroke on neuroimaging	−5.75	1.05	<0.01
Post-intervention	9.00	1.06	<0.01
6 months	9.96	1.06	<0.01
12 months	12.42	1.07	<0.01
Group × volume			<0.01
ASAP * log planimetric vol	−1.33	1.09	*A vs. U = 0.23* *A vs. D = <0.01*
DEUCC * log planimetric vol	4.17	0.93	*D vs. U = <0.01*
**CORTICAL** **+** **MIXED (*****N*** **=** **125)**			
Intercept	−7.22	2.13	<0.01
Age (years)	−0.09	0.04	0.02
Log planimetric volume (mL)	0.51	0.70	0.47
Treatment group (vs. UCC)			0.01
ASAP	1.81	2.23	*A vs. U = 0.42* *A vs. D = 0.03*
DEUCC	6.92	2.42	*D vs. U = <0.01*
Old stroke on neuroimaging	1.24	1.56	0.43
Post-intervention	8.06	2.57	<0.01
6 months	8.26	2.53	<0.01
12 months	11.00	2.58	<0.01
Group × volume			0.06

*Group effects are presented for omnibus, followed by comparisons; UCC is comparison for coefficeints (Change in UE-FM). A, ASAP; ASAP, Accelerated Skill Acquisition Program; D, DEUCC; DEUCC, Dose Equivalent Usual and Customary Care; U, UCC; UCC, Usual and Customary Care*.

#### Age and Prior Stroke on Neuroimaging

For all models, age predicted change in UE-FM score, with each additional year being associated with a 0.09–0.15 point lower increase in UE-FM scores, such that a 60 year old would recover ~1 point less than a 50 year old after a year. Old stroke was a statistically significant predictor in all models except the cortical + mixed subgroup, with participants achieving, on average 3.6–5.8 points less recovery in UE-FM. Thus, prior radiographic stroke on neuroimaging was the strongest predictor of recovery in most models.

#### Effects of Lesion Volume and Treatment Group

For the entire sample (*N* = 297), the best model showed that greater stroke volume was associated with slightly less recovery on UE-FM (−2.3 ±1.4 per mL volume, *P* = 0.01); there was no effect for treatment group assignment. There was a trend toward a treatment group by volume interaction that did not reach significance (*P* = 0.09).

For the subcortical subsample (*N* = 172), greater stroke volume was associated with substantially less recovery on UE-FM (−49.4 ± 1.9 per mL volume, *P* < 0.01). There was less recovery in the ASAP and DEUCC groups vs. UCC (−2.99 ± 1, *P* < 0.01 and −2.6 ± 1, *P* = 0.01, respectively), possibly suggesting that an increased dose of UE training is related to less recovery from subcortical stroke. This treatment group affect was modulated by lesion volume, with an association between larger lesion volumes and better response to DEUCC than ASAP or UCC.

The cortical + mixed subgroup (*N* = 125) showed no effect for volume, an association between DEUCC and more UE-FM recovery (6.92 ± 2.4, *P* < 0.01), and a trend toward a group by volume interaction that nearly reached significance (*P* = 0.06).

In summary, greater lesion volume was related to less recovery from motor impairment as measured by the UE-FM in participants with strokes in the overall sample, and particularly in those with subcortical stroke. Treatment group assignment was associated with recovery in select populations. Specifically, participants with subcortical stroke experienced slightly less recovery with increased dose of therapy (ASAP or DEUCC) whereas those with cortical + mixed lesions experienced more recovery with a higher dose of usual and customary therapy (DEUCC).

## Discussion

Many in the stroke rehabilitation community believe that lesion features play an important role in the potential for motor recovery and response to therapy after stroke (24). The ICARE trial size, three arm design, and access to clinical imaging provided an unprecedented opportunity to explore this belief. We showed that within the ICARE trial sample, which included patients with mild-moderate upper limb impairment in the subacute phase, the presence of old stroke on neuroimaging and lesion volume correlated with recovery from motor impairment. Further, we found that the type of motor training relates to recovery differently depending on lesion location. These findings are important for clinical trial design because they suggest that more refinement of trial participants based on neuroimaging criteria may be useful. These data provide new evidence that in the clinical trial setting, all types of strokes should not be treated as a unitary injury and that stratification by lesion characteristics may be necessary.

### Prior Radiographic Stroke

That participants with an old stroke on neuroimaging achieved less recovery from upper extremity impairment, particularly those with subsequent subcortical stroke, supports and extends the findings of the LEAPS trial. In LEAPS, a study of lower limb function (i.e., walking) in subacute stroke, evidence of prior stroke on neuroimaging led to less recovery of walking speed (14). The LEAPS investigators, however, did not analyze whether old strokes were particularly impactful in subcortical injury. Subcortical structures, such as the basal ganglia, internal capsule, and thalamus, are highly interconnected with multiple cortical areas, and injury to subcortical white matter tracts are known to interfere with interhemispheric functional connectivity in this distributed motor network (25). Patients who achieve excellent recovery >6 mo. after subcortical stroke show alterations in functional connectivity in multiple resting-state networks distributed throughout the brain (26, 27). Therefore, a possible explanation for our findings is that injury in the form of old stroke impairs network reserves, thereby reducing or exhausting the potential for activity-dependent neural plasticity in the setting of a subsequent subcortical stroke. There is some evidence for this dual hit theory in rodent stroke models, where small individual lesions to the sensorimotor cortex or dorsolateral striatum are clinically silent, but the combination of the two lesions causes motor impairment (28). Far fewer participants reported prior stroke by clinical history than the number identified radiographically, suggesting that many old strokes were clinically silent yet still associated with less recovery. The impact of prior radiographic stroke in the overall sample and in those with subcortical lesions (3.6 and 5.8 pts less recovery on UE-FM, respectively) is around the lower range of the reported minimum clinically important difference for the UE-FM (4.25–7.25 pts) (29). These findings could suggest a need to balance patients with prior radiographic stroke across treatment groups in future clinical trials to account for this important factor.

### Lesion Location/Volume

We found lesion location and volumes were associated with recovery, and thus future investigators may wish to account for them in future trial design. We predicted that stroke location would be an important determinant of stroke recovery based on emerging evidence linking the integrity of the CST with motor recovery following stroke (4, 5). While the effect of stroke lesion volume in the overall sample was small, we found a striking relationship in those with subcortical stroke. In this subgroup, increasing lesion volume by a single mL was related to nearly 50 points less recovery in UE-FM. This dramatic result does not seem physiologically plausible and likely reflects some mathematical artifact created by the mixed models and conversion of lesion volumes back from log scale; such large differences in recovery were rarely observed between ICARE participants. Nonetheless, this finding highlights how important small changes in lesion volume within the subcortical region can be with regard to motor recovery. We suspect this relationship results from the anatomy of the CST, and that larger volume strokes within the subcortical region cause more CST injury than those in cortical regions. A prior study showed a similar interaction between stroke volume and recovery within the subcortical stroke subgroup (3). We found that the impact of stroke volume on ΔUE-FM in patients with lesions centered in the anterior choroidal and basilar regions of our templates did not reach statistical significance, which seems counterintuitive considering we chose to study strokes centered in the ACH and basilar territories as a proxy for CST injury. We suspect that either lesions centered in the ACH and basilar territories are a poor surrogate for degree of CST injury in our template-based approach, or that concomitant injury to structures adjacent to the CST such as the basal ganglia are also important determinants of recovery. Indeed, the LEAPS trial investigators (14) and others (30), found reduced recovery in those with basal ganglia involvement.

### Response to Therapy

We found an association between certain lesion features and response to different types of therapy. Compared to UCC, subcortical stroke participants receiving a higher dose of UE training (ASAP or DEUCC) achieved 2–3 points less recovery on the UE-FM. In contrast, those with cortical or mixed lesions receiving DEUCC experienced nearly 7 points more recovery on the UE-FM in comparison to UCC; there was no difference between those receiving ASAP and UCC. Furthermore, stroke lesion volume moderated the effects of DEUCC, but not ASAP for the subcortical participants. There are mixed findings in the literature regarding lesion features and response to therapy, with some showing no effect of lesion location (31, 32) and others demonstrating less response to therapy in participants with lesions that injure the CST (7, 9, 33). Overall, our findings suggest that lesion location and volume could have an effect on response to therapy, however, the effect size was small for the subcortical group and seemingly heterogeneous between groups (therapy dose was important for subcortical lesions whereas therapy content and dose were important factors for participants with cortical + mixed lesions). Our finding of less recovery with higher doses of therapy in the subcortical group should be interpreted with caution. We are unaware of any other studies suggesting a negative response to therapy in the subacute phase and do not recommend limiting therapy to these patients. More robust, prospective studies are needed to gauge whether true associations exist between response to therapy and particular stroke lesion locations and volumes.

### Study Limitations

The ICARE population was limited to those with mild-moderate upper limb impairment and subacute stroke; we do not argue that these findings generalize to clinical populations. Our findings may not apply to more severely affected individuals or those studied in the acute or chronic time frame. There was a wide range of time since stroke when therapies were initiated in ICARE. We found a trend toward less recovery in participants with leukoaraiosis that did not reach statistical significance. Similar to the LEAPS trial investigators, our sample size may have been too small to determine whether leukoaraiosis impacts motor recovery. MRI/CT was not available on all randomized study participants and the UE-FM was not the primary outcome measure. Other measures of recovery that capture the ability to perform activities of daily living rather than motor impairment were not analyzed. Small, subcortical lesions are not well-visualized on CT and therefore the missing data from the remaining 64 participants is likely skewed toward these smaller lesions. Studying lesions centered in the ACH and basilar vascular territory regions may be a poor proxy for CST injury. We chose this method due to the lack of available diffusion tensor imaging from the clinical scans. We used a *p*-value of 0.10 to represent statistical significance in the mixed models, reflecting the exploratory nature of this secondary analysis. Finally, we did not have access to research grade images, which limited not only the kind of information, but the quality of information that we could use for these analyses. However, given this limitation, the exploratory analyses reported here may well serve to inform future prospective studies.

## Conclusions

We found that lesion volume in those with subcortical stroke and neuroimaging evidence of one or more old strokes were associated with less motor recovery in participants with mild-moderate upper limb impairment enrolled in a stroke rehabilitation trial. These findings corroborate and extend those of prior investigators (3, 6, 14) and suggest that future trialists may want to stratify study participants based on evidence of prior radiographic stroke. Lesion location may modulate response to different types of therapy, but the effects are small and heterogeneous, suggesting the need for well-designed, hypothesis-driven, prospective studies moving forward.

## Data Availability

The datasets generated for this study are available on request to the corresponding author.

## Ethics Statement

This study was carried out in accordance with the recommendations of the institutional review boards at Emory University, Georgetown University, and the University of Southern California with written informed consent from all subjects. All subjects gave written informed consent in accordance with the Declaration of Helsinki. The protocol was approved by the institutional review boards at each respective site.

## Author Contributions

ME and AD conceived the study. LD, CP, and CL performed statistical analysis. ME, MN, SW, CW, and AD were involved in data acquisition. All authors performed data interpretation. ME wrote the first draft of the manuscript. All authors contributed to manuscript revision, read and approved the submitted version.

### Conflict of Interest Statement

The authors declare that the research was conducted in the absence of any commercial or financial relationships that could be construed as a potential conflict of interest.
