# Supplementary material for: Reduced Upper Limb Recovery in Subcortical Stroke Patients With Small Prior Radiographic Stroke
Source: Front Neurol. 2019 May 8;10:454. doi: 10.3389/fneur.2019.00454 (PMC6517555; doi:10.3389/fneur.2019.00454)
Supplement: Supplementary file 1 [file Data_Sheet_1.PDF]

Supplementary Material

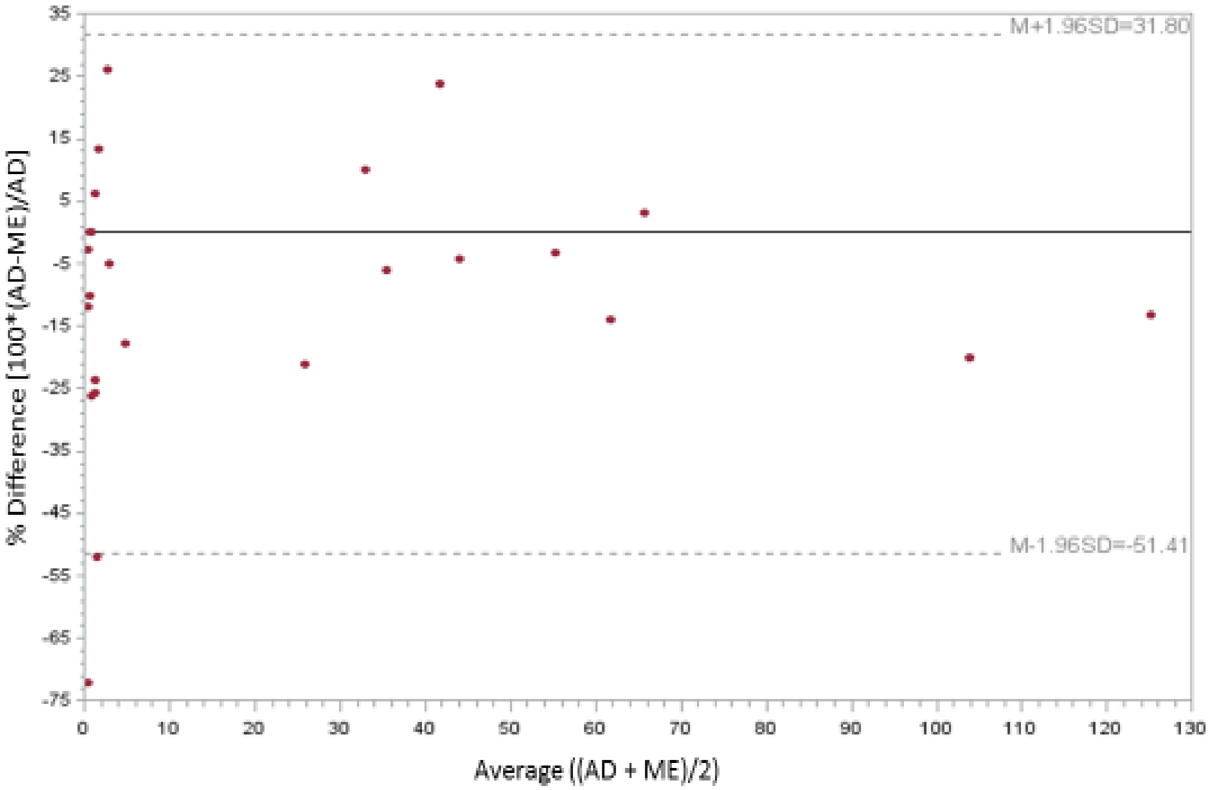

Supplementary Figure 1. Bland-Altman plot for planimetric volume between two raters AD and ME including 95% confidence intervals.
